# Therapeutic Effect of Low Doses of Acenocoumarol in the Course of Ischemia/Reperfusion-Induced Acute Pancreatitis in Rats

**DOI:** 10.3390/ijms18040882

**Published:** 2017-04-21

**Authors:** Zygmunt Warzecha, Paweł Sendur, Piotr Ceranowicz, Jakub Cieszkowski, Marcin Dembiński, Ryszard Sendur, Joanna Bonior, Jolanta Jaworek, Tadeusz Ambroży, Rafał Olszanecki, Beata Kuśnierz-Cabala, Kaczmarzyk Tomasz, Romana Tomaszewska, Artur Dembiński

**Affiliations:** 1 Department of Physiology, Faculty of Medicine, Jagiellonian University Medical College, 16 Grzegórzecka St., 31-531 Cracow, Poland; mpwarzec@cyf-kr.edu.pl (Z.W.); jakub.cieszkowski@uj.edu.pl (J.C.); ryszard.sendur@uj.edu.pl (R.S.); mpdembin@cyf-kr.edu.pl (A.D.); 2 The University Hospital in Cracow, 31-531 Cracow, Poland; p.send@interia.pl; 3 Second Department of General Surgery, Faculty of Medicine, Jagiellonian University Medical College, 31-531 Cracow, Poland; mpmdembi@cyf-kr.edu.pl; 4 Department of Medical Physiology, Faculty of Health Sciences, Jagiellonian University Medical College, 31-531 Cracow, Poland; joanna.bonior@uj.edu.pl (J.B.); jolanta.jaworek@uj.edu.pl (J.J.); 5 Department of Theory of Sport and Kinesiology, Faculty of Physical Education and Sport, University of Physical Education, 31-571 Cracow, Poland; tadek@ambrozy.pl; 6 Department of Pharmacology, Faculty of Medicine, Jagiellonian University Medical College, 31-531 Cracow, Poland; rafal.olszanecki@uj.edu.pl; 7 Department of Clinical Biochemistry, Faculty of Medicine, Jagiellonian University Medical College, 31-531 Cracow, Poland; mbkusnie@cyf-kr.edu.pl; 8 Department of Oral Surgery, Faculty of Medicine, Jagiellonian University Medical College, 31-531 Cracow, Poland; tomasz.kaczmarzyk@uj.edu.pl; 9 Department of Pathology, Faculty of Medicine, Jagiellonian University Medical College, 31-531 Cracow, Poland; romana.tomaszewska@uj.edu.pl

**Keywords:** acute pancreatitis, acenocoumarol, pancreatic blood flow, interleukin-1β, d-dimer, lipase, amylase

## Abstract

Intravascular activation of coagulation is observed in acute pancreatitis and is related to the severity of this inflammation. The aim of our study was to evaluate the impact of acenocoumarol therapy on the course of acute pancreatitis induced in male rats by pancreatic ischemia followed by reperfusion. Acenocoumarol at a dose of 50, 100, or 150 µg/kg/dose was administered intragastrically once a day, starting the first dose 24 h after the initiation of pancreatic reperfusion. Results: Histological examination showed that treatment with acenocoumarol reduces pancreatic edema, necrosis, and hemorrhages in rats with pancreatitis. Moreover, the administration of acenocoumarol decreased pancreatic inflammatory infiltration and vacuolization of pancreatic acinar cells. These findings were accompanied with a reduction in the serum activity of lipase and amylase, concentration of interleukin-1β, and plasma d-Dimer concentration. Moreover, the administration of acenocoumarol improved pancreatic blood flow and pancreatic DNA synthesis. Acenocoumarol given at a dose of 150 µg/kg/dose was the most effective in the treatment of early phase acute pancreatitis. However later, acenocoumarol given at the highest dose failed to exhibit any therapeutic effect; whereas lower doses of acenocoumarol were still effective in the treatment of acute pancreatitis. Conclusion: Treatment with acenocoumarol accelerates the recovery of ischemia/reperfusion-induced acute pancreatitis in rats.

## 1. Introduction

Coagulation is one of the mechanisms of hemostasis. Its activation causes clot formation to prevent blood loss from a damaged vessel. Coagulation disorders may take the form of insufficient or excessive clotting. Insufficient clotting can result in bleeding or hemorrhage; whereas excessive activation of clotting can lead to thrombosis and/or disseminated intravascular coagulation (DIC). Moreover, coagulation leads to the activation of inflammatory processes. Created during coagulation, thrombin and the complex of tissue factor plus active factor VII can stimulate protease activated receptors (PARs) [1,2]. This in turn can induce the expression of adhesion molecules leading to leukocyte-mediated injury [3,4]. Moreover, thrombin activates the production of monocyte chemotactic protein-1 (MCP-1) and pro-inflammatory interleukin-6 in fibroblasts, epithelial cells, and mononuclear cells [5]. The inflammatory response is also induced by the CD40 ligand released from activated platelets. The CD40 ligand stimulates the synthesis of the tissue factor and increases the level of pro-inflammatory interleukin-6 and interleukin-8 [3,6].

On the other hand, the relationship between coagulation and inflammation is bidirectional and is based on positive feedback. Activation of the coagulation induces inflammation, but at the same time the development of the inflammatory process activates the clotting cascade. Pro-inflammatory cytokines play an important role in the activation of coagulation by increasing expression of the tissue factor on monocytes and endothelium [6]. Moreover, inflammation reduces the activity of natural anticoagulants and inhibits fibrinolysis [3,4].

Coagulative disorders are also known to occur in acute pancreatitis and are related to the severity of this disease. In mild acute pancreatitis, the scattered intravascular thrombosis is observed in local pancreatic circulation; whereas severe acute pancreatitis may lead to the development of disseminated intravascular coagulation (DIC) [7,8,9,10]. Measurement of disseminated intravascular coagulation parameters is useful in the assessment of acute pancreatitis severity and has a prognostic value [9,10].

Previous experimental and clinical studies have shown the protective and therapeutic effect of heparin in acute pancreatitis [11,12,13,14,15,16]. Treatment with heparin has been also found to be effective in the prevention of pancreatitis-evoked encephalopathy [17]. Moreover, treatment with recombinant human soluble thrombomodulin seems to be useful in preventing walled-off necrosis in patients with severe acute pancreatitis [18].

In harmony with those findings is the observation that the inhibition of coagulation by pretreatment with low doses of acenocoumarol, a vitamin K antagonist, exhibits a protective effect on the pancreas and inhibits the development of acute pancreatitis induced by ischemia/reperfusion [19] or cerulein [20]. However, from a clinical point of view, it is more important to determine whether the administration of acenocoumarol after the development of acute pancreatitis exhibits any therapeutic effect on this disease. Therefore, the aim of this study was to answer the above stated question.

## 2. Results

### 2.1. International Normalized Ratio (INR) Value

**Figure 1:**
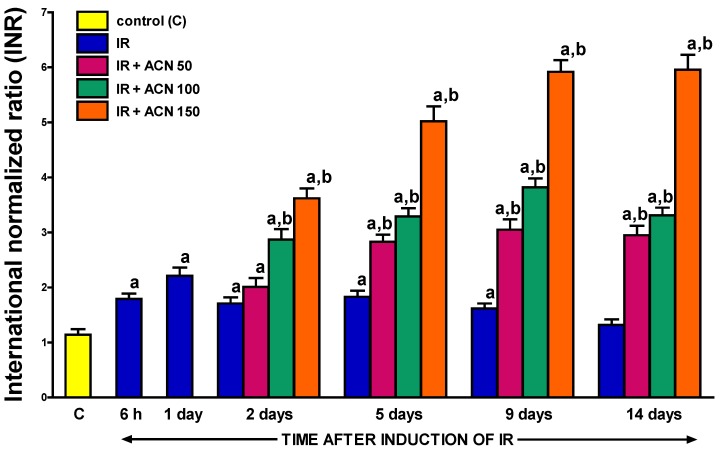
Influence of the treatment with acenocoumarol (ACN) on the prothrombin time expressed as the international normalized ratio (INR) in the course of ischemia/reperfusion-induced acute pancreatitis (IR). ACN was given at a dose of 50, 100, or 150 µg/kg/day (ACN 50, ACN 100, or ACN 150). Mean ± SEM. *n* = 8 rats in each experimental group. ^a^ *p* < 0.05 compared to control rats without induction of acute pancreatitis (C), ^b^ *p* < 0.05 compared to rats with IR without treatment with ACN at the same time of observation.

In control saline-treated sham-operated rats without induction of acute pancreatitis, the prothrombin time measured as the international normalized ratio (INR) reached a value of 1.14 ± 0.10 (Figure 1). Induction of acute pancreatitis by pancreatic ischemia followed by reperfusion led to an increase in INR and this effect was statistically significant between the 6th hour and 9th day of pancreatic reperfusion.

Treatment with acenocoumarol after the induction of acute pancreatitis additionally increased INR. In the case of acenocoumarol administered at a dose of 100 or 150 µg/kg/day, this effect reached a statistical significance on the second day of pancreatic reperfusion (1 day after the first dose of acenocoumarol). In the case of acenocoumarol administered at a dose of 50 µg/kg/day, this effect was statistically insignificant until the 5th day of reperfusion. Nine days after the start of pancreatic reperfusion, in rats treated with acenocoumarol given at a dose of 50, 100, or 150 µg/kg/day, the INR reached a value of 3.05, 3.82 or 5.92, respectively. At the 14th day of pancreatic reperfusion, the effects of treatment with acenocoumarol on the INR value in rats with ischemia/reperfusion-induced pancreatitis were similar to those observed at the 9th day of pancreatic reperfusion (Figure 1).

### 2.2. Histological Examination

**Figure 2:**
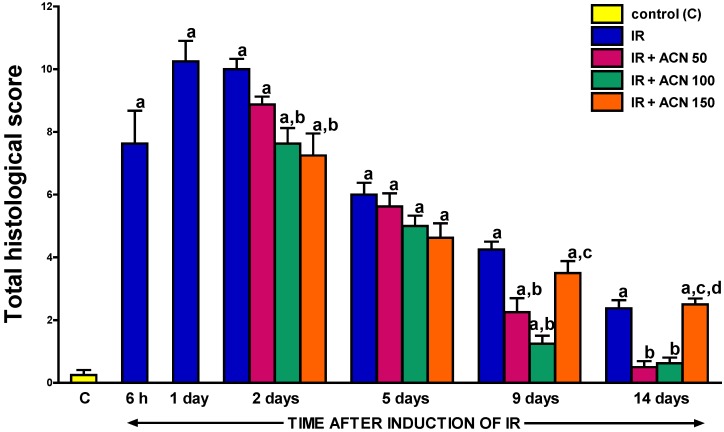
Influence of the treatment with acenocoumarol (ACN) on the total histological score of pancreatic damage in the course of ischemia/reperfusion-induced acute pancreatitis (IR). ACN was given at a dose of 50, 100, or 150 µg/kg/day (ACN 50, ACN 100, or ACN 150). Mean ± SEM. *n* = 8 rats in each experimental group. ^a^ *p* < 0.05 compared to control rats without induction of acute pancreatitis (C), ^b^ *p* < 0.05 compared to rats with IR without treatment with ACN at the same time of observation; ^c^ *p* < 0.05 compared to IR + ACN 100 at the same time of observation; ^d^ *p* < 0.05 compared to IR + ACN 50 at the same time of observation.

Pancreatic ischemia followed by reperfusion induced severe acute pancreatitis in all rats subject to this procedure. Macroscopic examination of the lung, liver, and kidney did not show any injury of those organs in the course of ischemia/reperfusion-induced pancreatitis. Maximal histological damage of pancreatic tissue was observed between the 1st and 2nd day of pancreatic reperfusion (Table 1, Figure 2). One day after the start of pancreatic reperfusion, moderate or severe inter- and intralobular edema was accompanied by moderate perivascular or abundant diffuse leukocyte infiltrations of the pancreatic tissue. The percentage of acinar cells with vacuolization was between less than 25% to 50% of these cells. Necrosis ranged from less than 15% to 35% of acinar cells. Moreover, 3 to 5 foci of hemorrhage were observed in each slide (Table 1, Figure 2). On subsequent days, the spontaneous regeneration of pancreatic tissue was observed. At the 14th day of pancreatic reperfusion, histological signs of pancreatic tissue damage were limited to a slight interlobular edema and minimal inflammatory perivascular infiltration. Vacuolization of acinar cells ranged between 0 to less than 25% of those cells.

**Figure 3:**
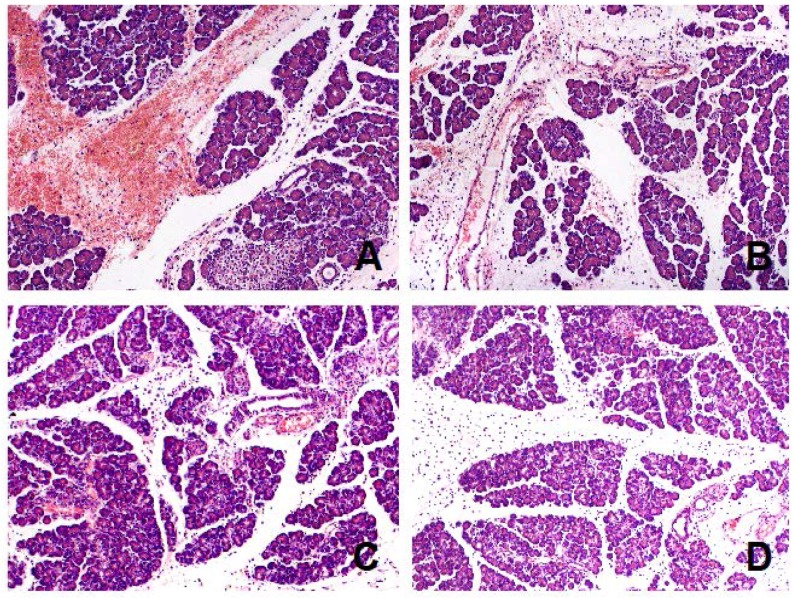
Representative morphological features of the pancreas observed after two-day pancreatic reperfusion in the course of ischemia/reperfusion-induced acute pancreatitis. (**A**) rats treated with saline; (**B**) rats treated with acenocoumarol given at a dose of 50 µg/kg/day; (**C**) rats treated with acenocoumarol given at a dose of 100 µg/kg/day; (**D**) rats treated with acenocoumarol given at a dose of 150 µg/kg/day. Hematoxylin-eosin counterstain. Original magnification 200×.

**Figure 4:**
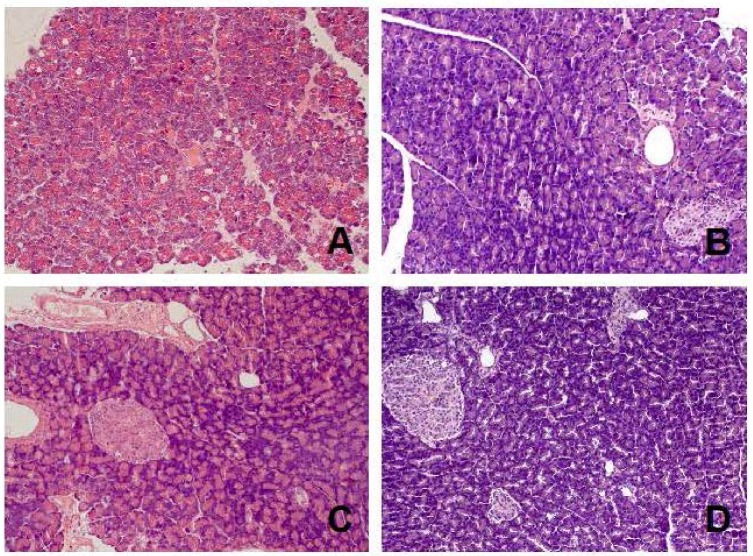
Representative morphological features of the pancreas observed after fourteen-day pancreatic reperfusion in the course of ischemia/reperfusion-induced acute pancreatitis. (**A**) rats treated with saline; (**B**) rats treated with acenocoumarol given at a dose of 50µg/kg/day; (**C**) rats treated with acenocoumarol given at a dose of 100 µg/kg/day; (**D**) rats treated with acenocoumarol given at a dose of 150 µg/kg/day. Hematoxylin-eosin counterstain. Original magnification 200×.

Treatment with acenocoumarol accelerated pancreatic regeneration in the course of ischemia/reperfusion-induced acute pancreatitis and this effect depended on the dose of acenocoumarol (Table 1). The first signs of pancreatic histology improvement were observed 1 day after the administration of the first dose of acenocoumarol. (Table 1). In the case of acenocoumarol given at a dose of 50 µg/kg/dose, one-day treatment with this coumarin only led to a slight decrease in the number of cells undergoing necrosis (Table 1, Figure 3). At the 5th day of pancreatic reperfusion, administration of acenocoumacol given at a dose of 50 µg/kg/dose had no effect on pancreatic morphology; whereas at the 9th day of reperfusion, the therapeutic effect of this dose of acenocoumarol was pronounced. A reduction in pancreatic edema, inflammatory infiltration, and number of hemorrhagic foci was observed. These effects led to a statistically significant reduction in the total histological score of pancreatic damage (Figure 2). At the 14th day of reperfusion, acenocoumarol administered at a dose of 50 µg/kg/day eliminated pancreatic edema and reduced inflammatory infiltration of pancreatic tissue. In this time of observation, the therapeutic effect of acenocoumarol given at a dose of 50 µg/kg/day was similar to that observed after acenocoumarol given at a dose of 100 µg/kg/day (Table 1, Figure 2 and Figure 4).

Acenocoumarol administered at a dose of 100 µg/kg/day caused the greatest improvement in the morphology of the pancreas. One-day treatment with this dose of acenocoumarol resulted in a decrease in pancreatic necrosis, inflammatory infiltration, and the number of foci of hemorrhage, leading to a significant reduction in the total histological score of pancreatic damage (Table 1, Figure 2 and Figure 3). The beneficial effect of acenocoumarol given at a dose of 100 µg/kg/day was also observed at the 5th, 9th, and 14th day of reperfusion as a reduction in pancreatic edema, inflammatory infiltration, and vacuolization of acinar cells (Table 1, Figure 2 and Figure 4). At the 9th day of reperfusion, acenocoumarol, given at a dose of 100 µg/kg/day, further reduced the number of hemorrhagic foci (Table 1).

In the case of acenocoumarol given at a dose of 150 µg/kg/day, a pronounced therapeutic effect was also observed at the 2nd and 5th day of pancreatic reperfusion, and this effect was similar to that observed after acenocoumarol given at a dose of 100 µg/kg/day (Table 1, Figure 2 and Figure 3). Starting from the 9th day of reperfusion, acenocoumarol given at a dose of 150 µg/kg/day was without any beneficial effect on pancreatic edema or inflammatory infiltration, Moreover, at the 9th and 14th day of pancreatic reperfusion, this dose of acenocoumarol resulted in an increase in the number of hemorrhagic foci in comparison to the value observed in the control group (Table 1). These effects led to a statistically significant increase in the total histological score of pancreatic damage at the 9th and 14th day of reperfusion in rats treated with acenocoumarol given at a dose of 150 µg/kg/day in comparison to pancreatic damage observed in rats treated with acenocoumarol given at a dose of 100 µg/kg/day (the 9th day) or rats treated with acenocoumarol given at a dose of either 50 or 100 µg/kg/day (the 14th day) (Figure 2).

### 2.3. Pancreatic Blood Flow

**Figure 5:**
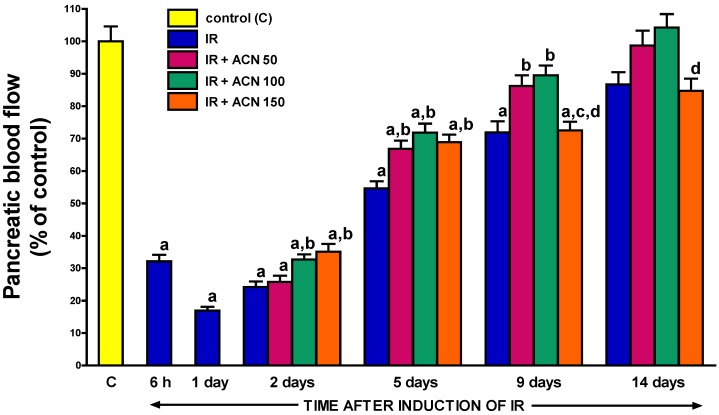
Influence of treatment with acenocoumarol (ACN) on pancreatic blood flow in the course of ischemia/reperfusion-induced acute pancreatitis (IR). ACN was given at a dose of 50, 100, or 150 µg/kg/day (ACN 50, ACN 100, or ACN 150). Mean ± SEM. *n* = 8 rats in each experimental group. ^a^ *p* < 0.05 compared to control rats without induction of acute pancreatitis (C), ^b^ *p* < 0.05 compared to rats with IR without treatment with ACN at the same time of observation; ^c^ *p* < 0.05 compared to IR + ACN 50 at the same time of observation; ^d^ *p* < 0.05 compared to IR + ACN 100 at the same time of observation.

Acute ischemia/reperfusion-induced pancreatitis resulted in an immediate dramatic reduction in pancreatic blood flow. After 6- and 24-h reperfusion, pancreatic blood flow was decreased by approximately 60% and 80%, respectively, as compared to the control group (Figure 5). This negative effect of ischemia/reperfusion-induced pancreatitis on pancreatic blood flow was not permanent and starting from the second day of reperfusion, this parameter spontaneously and gradually improved, reaching 87% of the control value on day 14. The administration of acenocoumarol affected pancreatic blood flow in the course of ischemia/reperfusion-induced pancreatitis. At the beginning of pancreatic reperfusion, all doses of acenocoumarol improved pancreatic blood flow. This effect was statistically significant at the second day of reperfusion after the administration of acenocoumarol at doses of 100 or 150 µg/kg/day. In the case of acenocoumarol given at a dose of 50 µg/kg/day, the statistically significant improvement of pancreatic blood flow was observed from the 5th day of pancreatic reperfusion. At the 9th day of reperfusion, acenocoumarol given at doses of 50 or 100 µg/kg/day significantly improved pancreatic blood flow in rats with ischemia/reperfusion-induced acute pancreatitis and pancreatic blood flow in these groups of animals reached a value similar to that observed in control rats without the induction of pancreatitis. In contrast, the administration of acenocoumarol at a dose of 150 µg/kg/day was without any beneficial effect on pancreatic blood flow at the 9th day of reperfusion (Figure 5) and pancreatic blood flow in this group of animals was statistically lower than that observed in rats treated with acenocoumarol at a dose of 100 µg/kg/day. A similar lack of a beneficial effect of acenocoumarol given at a dose of 150 µg/kg/day on pancreatic blood flow was also observed at the 14th day of pancreatic reperfusion (Figure 5).

### 2.4. Serum Level of Amylase and Lipase

**Figure 6:**
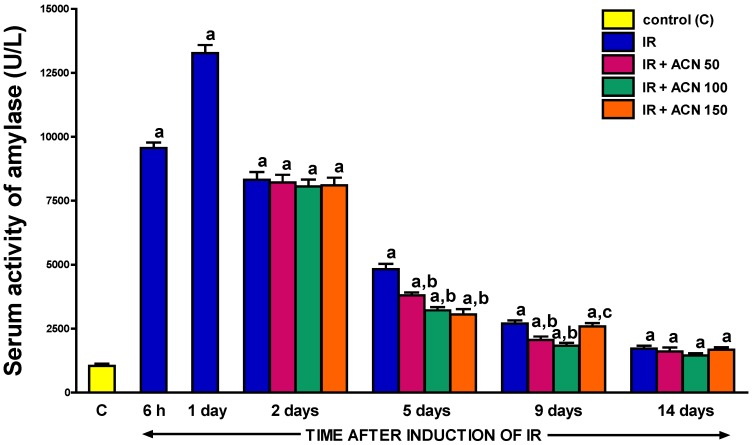
Influence of treatment with acenocoumarol (ACN) on serum activity of amylase in the course of ischemia/reperfusion-induced acute pancreatitis (IR). ACN was given at a dose of 50, 100, or 150 µg/kg/day (ACN 50, ACN 100, or ACN 150). Mean ± SEM. *n* = 8 rats in each experimental group. ^a^ *p* < 0.05 compared to control rats without induction of acute pancreatitis (C), ^b^ *p* < 0.05 compared to rats with IR without treatment with ACN at the same time of observation.

**Figure 7:**
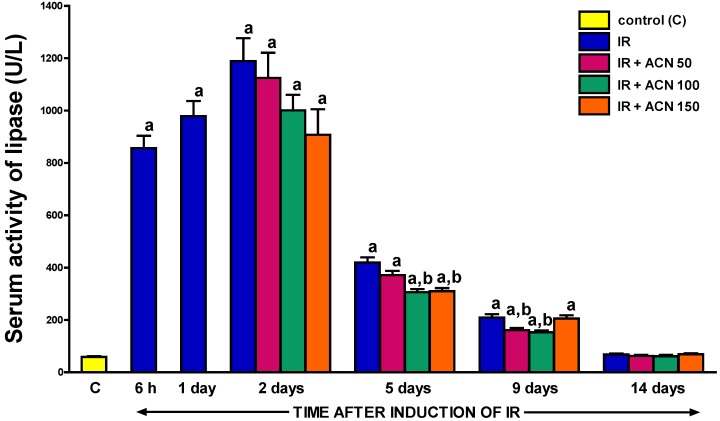
Influence of treatment with acenocoumarol (ACN) on serum activity of lipase in the course of ischemia/reperfusion-induced acute pancreatitis (IR). ACN was given at a dose of 50, 100, or 150 µg/kg/day (ACN 50, ACN 100, or ACN 150). Mean ± SEM. *n* = 8 rats in each experimental group. ^a^ *p* < 0.05 compared to control rats without induction of acute pancreatitis (C), ^b^ *p* < 0.05 compared to rats with IR without treatment with ACN at the same time of observation.

As demonstrated in Figure 6 and Figure 7, the development of acute ischemia/reperfusion-induced pancreatitis led to an increase in serum activity of amylase and lipase. Maximal serum activity of amylase was observed after one-day reperfusion of the pancreas. At that time, the activity of amylase was almost thirteen times greater than the value in control animals without induction of acute pancreatitis (Figure 6). The greatest activity of lipase was observed after a two-day reperfusion, reaching a value of more than twenty times greater than in the control group (Figure 7). Then serum activity of pancreatic enzymes spontaneously and gradually came down. The level of enzymes close to that in the control group was achieved two weeks following the start of pancreatic reperfusion. Treatment with acenocoumarol accelerated the normalization of serum amylase activity. This effect appeared for the first time at the 5th day of reperfusion. The effect of all doses of acenocoumarol was statistically significant; however, the highest dose of 150 µg/kg/day was the most effective. Four days later, low doses of acenocoumarol, 50 and 100 µg/kg/day, were still able to reduce the serum level of amylase, whereas the highest dose of acenocoumarol, 150µg/kg/day, was without a notable effect on this parameter. Two weeks after the start of reperfusion, treatment with any dose of acenocoumarol failed to significantly affect the serum activity of amylase (Figure 6).

A similar effect of treatment with acenocoumarol was observed in the case of the serum activity of lipase (Figure 7). The first marked impact of acenocoumarol administration was observed at the 5th day of pancreatic reperfusion. Acenocoumarol given at doses of 100 or 150 µg/kg/day significantly reduced the pancreatitis-evoked increase in the serum activity of lipase. In contrast, acenocoumarol given at a dose of 50 µg/kg/day failed to markedly affect the serum activity of lipase in this time of observation (Figure 7). Nine days after the start of pancreatic reperfusion, administration of acenocoumarol given at low doses of 50 or 100 µg/kg/day, significantly reduced the serum activity of lipase; whereas acenocoumarol given at the highest dose, 150 µg/kg/day, did not significantly affect this parameter. Moreover, the serum activity of lipase in this group of animals was markedly higher than in rats treated with lower doses of acenocoumarol (Figure 7). At the 14th day of pancreatic reperfusion, the serum activity of lipase was low in all groups treated with saline or any dose of acenocoumarol and was similar to that observed in the control group without the induction of acute pancreatitis (Figure 7).

### 2.5. Serum Concentration of Interleukin-1β

**Figure 8:**
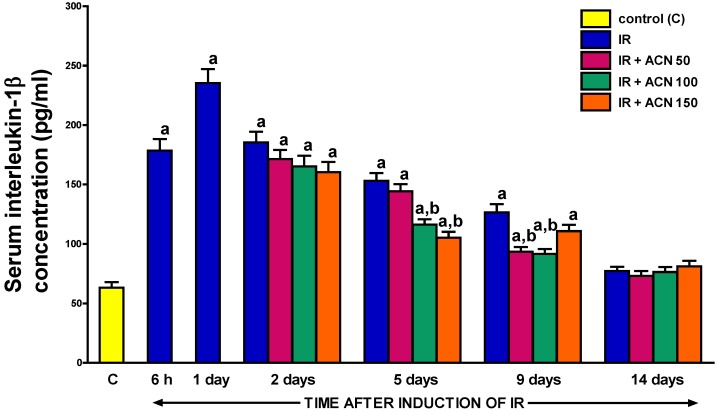
Influence of treatment with acenocoumarol (ACN) on serum concentration of interleukin 1β in the course of ischemia/reperfusion-induced acute pancreatitis (IR). ACN was given at a dose of 50, 100, or 150 µg/kg/day (ACN 50, ACN 100, or ACN 150). Mean ± SEM. *n* = 8 rats in each experimental group. ^a^ *p* < 0.05 compared to control rats without induction of acute pancreatitis (C), ^b^ *p* < 0.05 compared to rats with IR without treatment with ACN at the same time of observation.

The pattern of alterations in the serum concentration of pro-inflammatory interleukin-1β (Figure 8) in the course of ischemia/reperfusion-induced acute pancreatitis was similar to the type of changes in the serum activity of lipase. Following the initial increase in its value after the start of pancreatic reperfusion, with a maximal nearly 4-fold increase observed on day 1, there was observed a gradual normalization of interleukin-1β concentration throughout the study. Treatment with acenocoumarol accelerated that process and this effect was statistically significant between the 5th and 9th day of the experiment in the case of acenocoumarol given at a dose of 100 µg/kg/day (Figure 8). In the case of acenocoumarol given at a dose of 50 µg/kg/day, a statistically significant effect on the serum level of interleukin-1β was late and observed only at the 9th day of pancreatic reperfusion. In rats treated with acenocoumarol given at a dose of 150 µg/kg/day, a statistically significant reduction in serum interleukin-1β concentration was observed only at the 5th day of reperfusion. On day 14 of reperfusion, the serum concentration of interleukin-1β was similar in all groups of animals with acute pancreatitis regardless of the treatment and was similar to that observed in control animals without the induction of pancreatitis (Figure 8).

### 2.6. Pancreatic DNA Synthesis

**Figure 9:**
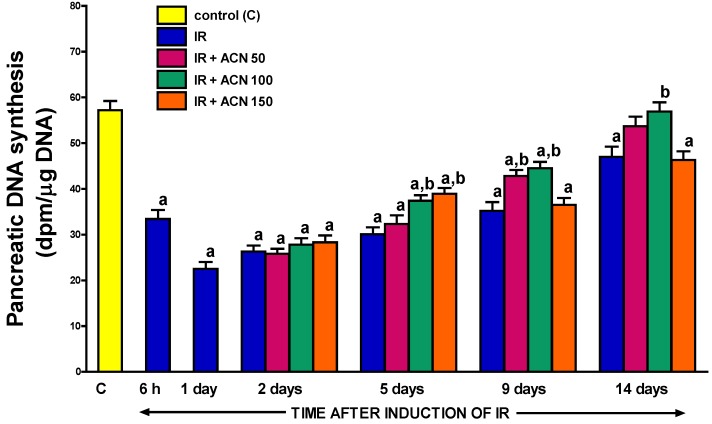
Influence of treatment with acenocoumarol (ACN) on pancreatic DNA synthesis in the course of ischemia/reperfusion-induced acute pancreatitis (IR). ACN was given at a dose of 50, 100, or 150 µg/kg/day (ACN 50, ACN 100, or ACN 150). Mean ± SEM. *n* = 8 rats in each experimental group. ^a^ *p* < 0.05 compared to control rats without induction of acute pancreatitis (C), ^b^ *p* < 0.05 compared to rats with IR without treatment with ACN at the same time of observation.

The induction of acute pancreatitis resulted in the reduction of pancreatic cell vitality and proliferation, measured as the rate of pancreatic DNA synthesis (Figure 9). Maximal reduction in that parameter was observed at the 1st day of pancreatic reperfusion, with subsequent gradual recovery during the next few days of observation. Treatment with acenocoumarol led to the partial reversion of the pancreatitis-evoked drop of pancreatic DNA synthesis and this effect was statistically significant between the 5th and 14th day of the study after administration of acenocoumarol at a dose of 100 µg/kg/day. The therapeutic effect of acenocoumarol given at a dose of 50 µg/kg/day was delayed and only visible at the 9th day of pancreatic reperfusion. Administration of acenocoumarol at a dose of 150 µg/kg/day resulted in a statistically significant improvement of pancreatic DNA synthesis at the 5th day of reperfusion, but later this dose of acenocoumarol was without a beneficial effect on this parameter (Figure 9).

### 2.7. Plasma d-Dimer Concentration

**Figure 10:**
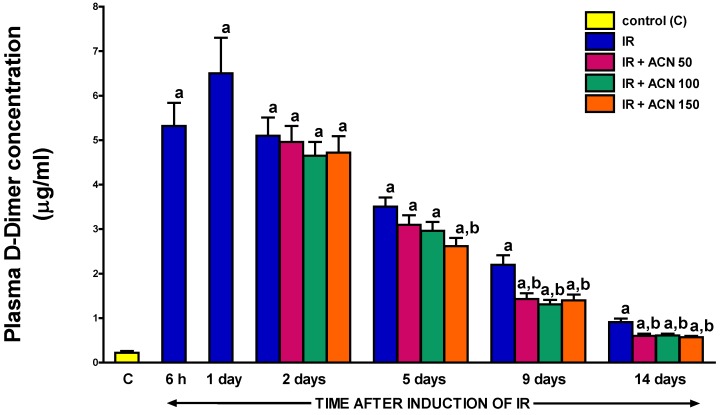
Influence of treatment with acenocoumarol (ACN) on plasma D-Dimer concentration in the course of ischemia/reperfusion-induced acute pancreatitis (IR). ACN was given at the dose of 50, 100, or 150 µg/kg/day (ACN 50, ACN 100, or ACN 150). Mean ± SEM. *n* = 8 rats in each experimental group. ^a^ *p* < 0.05 compared to control rats without induction of acute pancreatitis (C), ^b^ *p* < 0.05 compared to rats with IR without treatment with ACN at the same time of observation.

Acute ischemia/reperfusion-induced pancreatitis significantly increased the plasma concentration of d-Dimer, with the highest, almost a 30-fold increase, seen after one-day pancreatic reperfusion (Figure 10). Then the gradual spontaneous normalization of the plasma d-Dimer concentration was seen over time, beginning on day 2. Administration of acenocoumarol accelerated the reduction in d-Dimer and this effect was dependent on the dose of acenocoumarol. After treatment with acenocoumarol given at a dose of 150 µg/kg/day, a significant decrease in the d-Dimer concentration was apparent by day 5 of pancreatic reperfusion and lasted until the end of the observation time. In the case of acenocoumarol given at a dose of 100 µg/kg/day, a statistically significant reduction in the pancreatitis-evoked increase in the plasma d-Dimer concentration was observed between the 9th and 14th day of reperfusion. Acenocoumarol given at a dose of 50 µg/kg/day significantly affected the plasma d-Dimer concentration at the 9th and 14th day of pancreatic reperfusion (Figure 10).

## 3. Discussion

Our present study provided several important findings regarding the role of coagulation in acute ischemia/reperfusion-induced pancreatitis and the influence of treatment with acenocoumarol on the course of this inflammation. We observed that the development of ischemia/reperfusion-induced pancreatitis is associated with the activation of clotting. Six hours after the start of pancreatic reperfusion, the plasma d-Dimer concentration reached around a 24-fold higher value than in the control group. The maximal level of the d-Dimer concentration was found after a one-day pancreatic reperfusion and the spontaneous reduction in this parameter was observed in the subsequent days of observation. However, even two weeks after the induction of pancreatitis, the level of d-Dimer was higher than in the control group.

The above changes in plasma d-Dimer concentration were accompanied by an increase in INR. Those findings indicate that the course of ischemia/reperfusion-induced acute pancreatitis is associated with the activation of coagulation followed by subsequent fibrinolysis and consumptive coagulopathy. This observation is in harmony with previous studies showing that coagulative disorders are frequent in acute pancreatitis and the determination of coagulative parameters may be useful in the prediction of fatal outcome in patients with this disease [10,21].

Previous studies have shown that pretreatment with acenocoumarol inhibits the development of ischemia/reperfusion-, as well as, cerulein-induced acute pancreatitis [19,20]. However, it must be pointed out that patients with acute pancreatitis are usually seen in the hospital several hours or even days after the onset of this disease. For this reason, the preventive effect of acenocoumarol against the development of acute pancreatitis has only a limited clinical significance. Our current study showed that treatment with low doses of acenocoumarol after the development of acute pancreatitis accelerates the recovery in ischemia/reperfusion-induced pancreatitis. To the best of our knowledge, this is the first report that acenocoumarol exhibits a therapeutic effect in the course of this disease.

The beneficial effect of acenocoumarol in acute pancreatitis has been evidenced by the improvement of the morphological, biochemical, and functional parameters of the pancreatic condition. Administration of acenocoumarol after the induction of acute pancreatitis increased INR and reduced plasma d-Dimer concentration and those effects were associated with a faster normalization of pancreatic histology leading to earlier reduction and/or elimination of pancreatic edema and leukocyte inflammatory infiltration of the pancreatic tissue. These effects were accompanied by a reduction in pancreatic necrosis, vacuolization of acinar cells, and number of hemorrhagic foci. The therapeutic effect of acenocoumarol was dose-dependent. Acenocoumarol given at the highest dose of 150 µg/kg/dose was the most effective in the treatment of early phase acute pancreatitis. However later, acenocoumarol given at the highest dose failed to exhibit any beneficial effect on the pancreatic morphology. Moreover, at the 9 and 14th day of pancreatic reperfusion, acenocoumarol given at a dose of 150 µg/kg/dose resulted in an increase in the number of hemorrhagic foci in the pancreas. In contrast, lower doses of acenocoumarol, 50 and 100 µg/kg/dose, were less effective in early phase acute pancreatitis, but they were still effective at the end of the observation period.

The acenocoumarol-related reduction in inflammatory leukocyte infiltration of pancreatic tissue was in harmony with a decrease in the pancreatitis-evoked increase in the serum level of interleukin-1β. In acute pancreatitis, pro-inflammatory cytokines such as interleukin-1β, inerleukin-6, and tumor necrosis factor-α (TNF-α) are primary produced within the pancreas and subsequently within distant organs, developing systemic inflammatory response syndrome (SIRS) and multiple organ failure (MOF) in severe cases of this disease [22,23]. Interleukin-1β plays an essential role in the initiation and course of inflammatory processes. It exhibits direct pro-inflammatory activity, as well as stimulates the release of other members of the pro-inflammatory cytokine cascade [24,25]. The severity of AP is well-correlated with the production of pro-inflammatory cytokines [22]. Previous studies showed that the administration of the interleukin-1β receptor antagonist prevents a serum rise in interleukin-6 and TNF-α levels, and decreases the severity of acute pancreatitis [26]. It indicates that interleukin-1β plays an important role in the development and course of this disease.

In the current study, we found an increase in serum concentration of interleukin-1β after the development of ischemia/reperfusion-induced acute pancreatitis and this effect was followed by the spontaneous gradual normalization of the serum level of this pro-inflammatory cytokine over time. The above observation is in agreement with previous reports concerning the serum level of interleukin-1β in the course of acute pancreatitis [27,28]. A new important finding of our current study was the discovery that treatment with acenocoumarol led to a significant decrease in the serum interleukin-1β concentration and limitation of the severity of acute pancreatitis. Moreover, this observation is in line with previous reports showing that pretreatment with acenocoumarol before the induction of acute pancreatitis reduces the pancreatitis-evoked increase in the serum level of interleukin-1β [19,20]. These findings taken together indicate that the acenocoumarol-induced reduction in the serum level of interleukin-1β is involved in the protective and therapeutic effect of acenocoumarol in acute pancreatitis.

Further supporting evidence of the therapeutic effect of acenocoumarol in the course of ischemia/reperfusion-induced acute pancreatitis is related to the reversion of pancreatitis-evoked increases in the serum concentration of amylase and lipase. Normally, these enzymes are released by the pancreas into the duodenum. However, in acute pancreatitis, they are ectopically released into the interstitial space of the pancreas and hence into the circulatory system. For this reason, the serum levels of amylase and lipase are well-established indices of acute pancreatitis development and severity with high sensitivity and specificity [28,29]. Active pancreatic enzymes present in the interstitial space of the pancreas and circulation lead to local and remote organ injury, as well as up-regulate the expression of adhesion molecules on leukocytes and endothelial cells, leading to an increase in leukocyte-endothelial interaction and disturbance of organ microcirculation [30].

In our present study, the highest serum activity of amylase was observed after one-day reperfusion of the pancreas. At that time, the activity of amylase was almost thirteen-fold greater than the value in control animals without acute pancreatitis. In the case of lipase, maximal serum activity of this enzyme was observed after a two-day reperfusion, reaching a value of more than twenty times greater than in the control rats without acute pancreatitis. Then a spontaneous decrease in serum activity of amylase and lipase was observed throughout the study, starting on day 2 or 5, respectively. Treatment with acenocoumarol accelerated the normalization of serum pancreatic digestive enzyme activity. This effect was statistically significant between the 5th and 9th day of observation in the case of both enzymes tested.

Adequate blood flow through the organ microcirculation is necessary to provide and sustain oxygenation and nutrition at a cellular level. Clinical and experimental observations showed that pancreatic ischemia plays an essential role in the development of acute pancreatitis and progression of the severity of this disease [31,32,33,34]. Pancreatic ischemia/reperfusion injury may be a primary cause of acute pancreatitis [31,32,33], but also in acute pancreatitis caused by other, primary non-vascular mechanisms, the early disturbance of pancreatic circulation is observed [35,36]. The development of acute pancreatitis is associated with microvascular impairment of the gland with subsequent formation of thrombi in capillaries, activation of leukocytes, and the release of digestive enzymes and-pro-inflammatory cytokines [34,35]. These factors act locally in the pancreas, as well as going into the circulation which can result in systemic effects as systemic inflammatory response syndrome (SIRS), multiple organ failure (MOF) and ultimately death [22,25]. On the other hand, there are studies showing that the improvement of pancreatic blood flow inhibits the development of acute pancreatitis and accelerates recovery in this disease [37,38,39]. In agreement with previous reports, our current study confirmed a deleterious impact of acute pancreatitis on pancreatic perfusion. Maximal reduction of pancreatic blood flow by more than 80% was observed after one-day reperfusion. This blood flow restriction in the pancreas was temporary and starting from the second day of reperfusion, this parameter spontaneously and gradually improved reaching almost the control value at the last day of observation. Administration of acenocoumarol improved pancreatic blood flow in the course of ischemia reperfusion-induced pancreatitis. This effect was statistically significant at the second day of reperfusion after administration of acenocoumarol at higher doses of 100 or 150 µg/kg/day. Acenocoumarol given at a dose of 50 µg/kg/day was able to induce an improvement of pancreatic blood flow from the 5th day of reperfusion. Improvement of pancreatic blood flow was accompanied by an improvement of pancreatic morphology and reduction in biochemical markers of inflammation severity. At the 9th day of reperfusion, acenocoumarol given at doses of 50 or 100 µg/kg/day was still able to significantly improve pancreatic blood flow; whereas administration of acenocoumarol at a dose of 150 µg/kg/day was without any beneficial effect on pancreatic blood flow or pancreatic morphology at that time of observation. These data indicate that appropriate pancreatic blood flow is necessary for pancreatic recovery and the improvement of pancreatic blood flow is involved in the therapeutic effect of acenocoumarol in the course of acute pancreatitis.

Cell renewal is essential for the treatment of damaged organs. The cellular rate of DNA synthesis is an index of cell vitality and proliferation. Several previous studies have demonstrated that acute pancreatitis, irrespective of etiology, led to an initial reduction in pancreatic DNA synthesis followed by a subsequent increase in this parameter [40,41]. Our current observations confirmed this pattern in the ischemia/reperfusion-induced acute pancreatitis in which the severity of pancreatic injury was very pronounced and pancreatic cell proliferation was initially reduced by approximately 80%. Moreover, our current study showed that the administration of acenocoumarol after the onset of acute pancreatitis significantly accelerates the restoration of the appropriate rate of pancreatic DNA synthesis leading to the acceleration of recovery in this disease. This finding is additional evidence that acenocoumarol exhibits a therapeutic effect in acute pancreatitis and the mechanism of this effect involves the improvement of cell vitality and cell proliferation.

Our current study showed that the development of ischemia/reperfusion-induced acute pancreatitis leads to an increase in the INR value. Administration of acenocoumarol led to an additional increase in this parameter. These changes in INR may lead to an increase in risk of hemorrhage. However, intraabdominal hemorrhage was not observed during treatment with acenocoumarol in our present study. We only found the presence of foci of hemorrhage in histological examinations of the pancreas and low doses of acenocoumarol reduced the number of these lesions. Also, a small number of spontaneous animal deaths was observed only before the second day of pancreatic reperfusion. Later, when there was a marked increase in INR, further deaths among the animals were not found. Moreover, the most frequent cause of hemorrhage in the course of acute pancreatitis is not a decrease in coagulation, but the erosion of small vessels [42]. On the other hand, we used an animal model of acute pancreatitis. The course of acute pancreatitis in animal models resembles the course of this inflammation in humans, but it is not identical [43]. The anatomy and physiology of the pancreas in humans are different from the anatomy and physiology of the pancreas in rats. For this reason, our study suggests the potential usefulness of acenocoumarol in the treatment of acute pancreatitis, but further research is required before introducing this type of therapy in this disease.

## 4. Materials and Methods

### 4.1. Animals and Treatment

All studies followed an experimental protocol approved by the Committee for Research and Animal Ethics of the Jagiellonian University and the First Local Commission of Ethics for the Care and Use of Laboratory Animals in Cracow (Permit Number 4/2013 released on 16 January 2013).

Studies were carried out on 156 male Wistar rats weighing 250–270 g, which were housed in cages within a windowless colony room. We originally used 152 rats, but 4 animals died before the second day of pancreatic reperfusion and before intragastric treatment with saline or acenocoumarol. They died due to abdominal hemorrhage and ascites leading to circulatory insufficiency. For this reason, 4 additional rats were used to reach 8 observations in each experimental group and each endpoint of the studies. The temperature in the colony room was adjusted at 22 ± 1 °C with relative humidity of 50% ± 10%, and 12:12 h light:dark photoperiod. Following a one-week period of acclimation to their new environment, rats were randomly assigned to 5 experimental groups, as follows: (1) sham-operated saline-treated rats (control group); (2) rats treated with saline after the development of ischemia/reperfusion-induced acute pancreatitis; (3) rats treated with acenocoumarol, given at a dose of 50 µg/kg/day, after the development of ischemia/reperfusion-induced acute pancreatitis; (4) rats treated with acenocoumarol given at a dose of 100 µg/kg/day after the development of ischemia/reperfusion-induced acute pancreatitis; (5) rats treated with acenocoumarol given at a dose of 150 µg/kg/day after the development of ischemia/reperfusion-induced acute pancreatitis. The study was terminated after 6 h or 1, 2, 5, 9, or 14 days after the initiation of pancreatic reperfusion or sham-operation. Each experimental group in each time of observation consisted of 8 rats.

The surgical procedure for the induction of ischemia/reperfusion-induced pancreatitis was performed according to the method previously described [40]. Rats were fasted with free access to water for 24 h before the induction of acute pancreatitis or sham-operation. Before the surgery, animals were anesthetized with ketamine (Bioketan, Vetoquinol Biowet, Gorzów Wielkopolski, Poland) given intraperitoneally (i.p.) at a dose of 50 mg/kg. After a longitudinal laparotomy, the inferior splenic artery was clamped down by microvascular clips to induce pancreatic ischemia in the splenic region of this organ. Thirty min later, microvascular clips were removed to reach pancreatic reperfusion and the abdominal cavity was closed by sutures. In control sham-operated rats, after longitudinal laparotomy the mobilization of the pancreas was performed, without clamping any artery.

During the first two days after surgery, animals were monitored every 8 h and received tramadol (Poltram 100, Polpharma, Starogard Gdański, Poland) given subcutaneously (s.c.) at a dose of 1 mg/kg/dose to minimize pain and distress. Also, immediately after surgery, as well as 12 and 24 h later, all animals were injected s.c. with 10 mL of Ringer’s solution for the supply of fluids lost during surgery and postoperative period.

Treatment with saline or acenocoumarol was started 24 h after the beginning of pancreatic reperfusion. Saline or acenocoumarol (Acenocumarol WZF, Warszawskie Zakłady Farmaceutyczne Polfa S.A., Warsaw, Poland) were given intragastrically once daily until the day of killing the animals, which was the last day of treatment. Acenocoumarol was administered at doses of 50, 100, or 150 µg/kg/day because the earlier study [19] showed that those doses increased the international normalized ratio (INR) to a value between 2.5 and 3.5. This range of INR is recommended in most clinical situations associated with hypercoagulation [44].

### 4.2. Determination of Pancreatic Blood Flow

Rats were again anesthetized with ketamine at the each time of observation. After opening the abdomen and exposure of the pancreas, blood flow in the splenic region of the pancreas was assessed using a laser Doppler flowmeter (PeriFlux 4001 Master Monitor, Perimed AB, Järfälla, Sweden), as described previously in detail [45,46]. Each result was expressed as a percentage of a mean value obtained in control sham-operated saline-treated rats without the induction of acute pancreatitis.

### 4.3. Biochemical Analysis

Immediately after the assessment of pancreatic blood flow, samples of blood were withdrawn from the abdominal aorta. The prothrombin time expressed as the international ratio (INR) was assessed using Alere INRatio^®^ 2 PT/INR Monitoring Systems and Alere INRatio^®^ PT/INR Monitoring System Test Strips purchased from Alere San Diego, Inc., San Diego, CA, USA.

The level of plasma d-Dimer concentration was assessed on a coagulation analyzer BCS XP System (Simens Healthcare Diagnostics, Erlangen, Germany), using an immunoturbidimetric assay (Innovance d-Dimer Assay, Simens Healthcare GmbH, Marburg, Germany).

Serum activity of pancreatic digestive enzymes, lipase and amylase, was assessed on the Kodak Ectachem DT II System analyzer (Eastman Kodak Company, Rochester, NY, USA), using Lipa DT, and Amyl DT Slides (Vitros DT Chemistry System, Johnson & Johnson Clinical Diagnostic, Inc., Rochester, NY, USA).

The serum level of interleukin-1β (IL-1β) was determined using the Rat IL-1β Platinum Elisa purchased from Bender MedSystem GmbH, Vienna, Austria.

### 4.4. Determination of Pancreatic DNA Synthesis

After the blood withdrawal, the pancreas was carefully dissected out from its attachment to neighboring organs. Samples of pancreatic tissue were taken for study of the pancreatic DNA synthesis and histological examination. The rate of DNA synthesis was determined by the measurement of the incorporation of tritium labeled thymidine ([6-^3^H]-thymidine, 20–30 Ci/mmol, Institute for Research, Production and Application of Radioisotopes, Prague, Czech Republic) into pancreatic DNA, as described previously in detail [40,47]. The rate of DNA synthesis was expressed as a number of tritium atoms disintegrations per minute per microgram DNA (dpm/µg DNA).

### 4.5. Histological Examination of Pancreatic Damage

Samples of pancreatic tissue were fixed in 10% buffered formalin. Slides were stained with hematoxylin and eosin, and were examined by two pathologists who were unaware of the treatment given. As described previously [48], the following histological signs of pancreatic damage were examined (grading from 0 to 3):

1. pancreatic edema (0—no edema, 1—interlobar edema, 2—interlobar and moderate intralobular edema, 3—severe interlobula and intralobular edema);
2. leukocyte inflammatory infiltration (0—Absent, 1—scarce perivascular infiltration, 2—moderate perivascular and scarce diffuse infiltration, 3—abundant diffuse infiltration);
3. vacuolization of acinar cells (0—absent, 1—involving less than 25% of acinar cells, 2—involving from 25% to 50% acinar cells, 3—involving more than 50% of acinar cells);
4. necrosis of acinar cells (0—absent, 1—involving less than 15% of acinar cells, 2—involving from 15% to 35% acinar cells, 3—involving more than 35% of acinar cells);
5. hemorrhage (0—absent, 1—from 1 to 2 foci per slide, 2—from 3 to 5 foci per slide, 3—more than 5 foci per slide).

The results of the histological examination results have been shown in Table 1 as a predominant histological grading (mode) of pancreatic edema, inflammatory infiltration, vacuolization and necrosis of acinar cells, and hemorrhage in each experimental group. Moreover, we have presented the representative morphological features of the pancreas and total histological score of pancreatic damage calculated as the sum of degrees of pancreatic edema, inflammatory infiltration, vacuolization of acinar cells, necrosis of these cells, and the presence of hemorrhage.

### 4.6. Statistical Analysis

Statistical analysis was made by analysis of variance followed by Tukey’s multiple comparison test using GraphPadPrism (GraphPad Software, San Diego, CA, USA). Statistical analysis for each observation period was calculated separately. Results were presented as means ± SEM. Each experimental group consisted of eight animals. A difference with a *p* value of less than 0.05 was considered significant.

## 5. Conclusions

In conclusion, we can say that treatment with low doses of acenocoumarol reduces the severity of ischemia/reperfusion-induced acute pancreatitis in rats and accelerates recovery in this disease. These findings suggest that treatment with low doses of acenocoumarol may inhibit coagulative disorders in the course of acute pancreatitis and perhaps it can bring some benefits in the treatment of acute pancreatitis in humans. However, due to the potential risk of hemorrhage, further research is needed in this area.

## Figures and Tables

**Table 1:** Influence of the treatment with acenocoumarol (ACN) on pancreatic morphological signs of pancreatic damage in the course of ischemia/reperfusion-induced acute pancreatitis (IR). ACN was given at a dose of 50, 100 or 150 µg/kg/day (ACN 50, ACN 100, or ACN 150).

Experimental Groups		Edema (0–3)	Inflammatory Infiltration (0–3)	Vacuolization (0–3)	Necrosis (0–3)	Hemorrhages (0–3)
Control		0	0	0	0	0
I/R (6 h)		2–3	1–2	1	1	1–2
I/R (1 day)		2–3	3	1–2	1–2	2
I/R (2 days)	Saline	2	3	1	1–2	2
	ACN 50	2	3	1	1	2
	ACN 100	2	2–3	1	0–1	1–2
	ACN 150	2	2–3	1	0–1	1–2
I/R (5 days)	Saline	1–2	2	1	1	0–1
	ACN 50	1–2	2	1	1	0–1
	ACN 100	1	1–2	1	1	0–1
	ACN 150	1	1–2	1	1	0–1
I/R (9 days)	Saline	1	2	0–1	0	0–1
	ACN 50	0–1	1–2	0–1	0	0
	ACN 100	0–1	1	0	0	0
	ACN 150	1	2	0	0	1
I/R (14 days)	Saline	1	1	0–1	0	0
	ACN 50	0	0–1	0	0	0
	ACN 100	0	0–1	0	0	0
	ACN 150	1	1	0	0	0–1

Numbers represent the predominant histological grading in each experimental group.
