# Correction: Warzecha, Z., et al. Therapeutic Effect of Low Doses of Acenocoumarol in the Course of Ischemia/Reperfusion-Induced Acute Pancreatitis in Rats. *Int. J. Mol. Sci.* **2017**, *18*, 882

**DOI:** 10.3390/ijms20123086

**Published:** 2019-06-24

**Authors:** Zygmunt Warzecha, Paweł Sendur, Piotr Ceranowicz, Jakub Cieszkowski, Marcin Dembiński, Ryszard Sendur, Joanna Bonior, Jolanta Jaworek, Tadeusz Ambroży, Rafał Olszanecki, Beata Kuśnierz-Cabala, Kaczmarzyk Tomasz, Romana Tomaszewska, Artur Dembiński

**Affiliations:** 1 Department of Physiology, Faculty of Medicine, Jagiellonian University Medical College, 16 Grzegórzecka St., 31-531 Cracow, Poland; mpwarzec@cyf-kr.edu.pl (Z.W.); jakub.cieszkowski@uj.edu.pl (J.C.); ryszard.sendur@uj.edu.pl (R.S.); mpdembin@cyf-kr.edu.pl (A.D.); 2 The University Hospital in Cracow, 31-531 Cracow, Poland; p.send@interia.pl; 3 Second Department of General Surgery, Faculty of Medicine, Jagiellonian University Medical College, 31-531 Cracow, Poland; mpmdembi@cyf-kr.edu.pl; 4 Department of Medical Physiology, Faculty of Health Sciences, Jagiellonian University Medical College, 31-531 Cracow, Poland; joanna.bonior@uj.edu.pl (J.B.); jolanta.jaworek@uj.edu.pl (J.J.); 5 Department of Theory of Sport and Kinesiology, Faculty of Physical Education and Sport, University of Physical Education, 31-571 Cracow, Poland; tadek@ambrozy.pl; 6 Department of Pharmacology, Faculty of Medicine, Jagiellonian University Medical College, 31-531 Cracow, Poland; rafal.olszanecki@uj.edu.pl; 7 Department of Clinical Biochemistry, Faculty of Medicine, Jagiellonian University Medical College, 31-531 Cracow, Poland; mbkusnie@cyf-kr.edu.pl; 8 Department of Oral Surgery, Faculty of Medicine, Jagiellonian University Medical College, 31-531 Cracow, Poland; tomasz.kaczmarzyk@uj.edu.pl; 9 Department of Pathology, Faculty of Medicine, Jagiellonian University Medical College, 31-531 Cracow, Poland; romatom@mp.pl

We would like to submit this correction to our published paper [1]. The reason for the correction is an error in the histological image presented in the article. One histological image (Figure 4C in [1]) is incorrect and for this reason it should be replaced with the correct new figure, shown below (Figure 1).

The abovementioned error in the original article did not have a material impact on the final results and conclusions of our papers. We apologize to our readers for this inconvenience. 

## Figures and Tables

**Figure 1:**
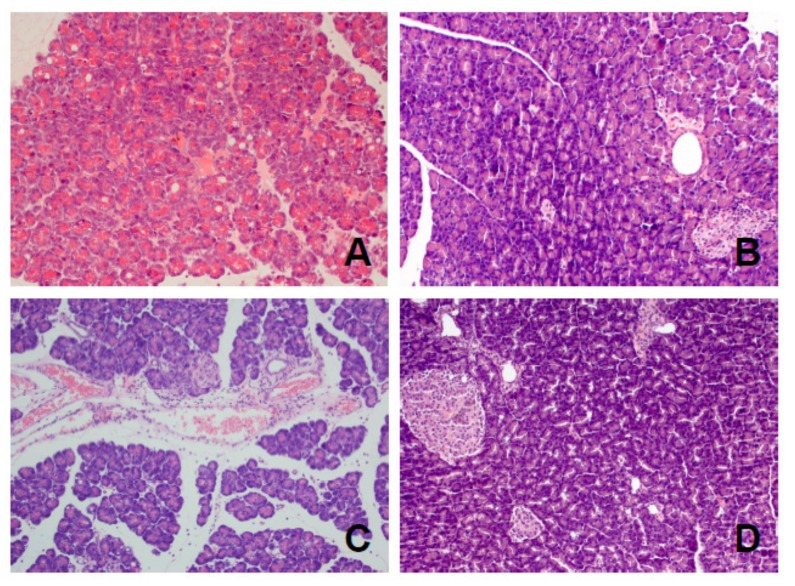
Representative morphological features of the pancreas observed after 14 day pancreatic reperfusion in the course of ischemia/reperfusion-induced acute pancreatitis. (**A**) Rats treated with saline; (**B**) rats treated with acenocoumarol given at a dose of 50 µg/kg/day; (**C**) rats treated with acenocoumarol given at a dose of 100 µg/kg/day; (**D**) rats treated with acenocoumarol given at a dose of 150 µg/kg/day. Hematoxylin-eosin counterstain. Original magnification 200×.
